# Nutritional Rehabilitation and Medical Stabilization of Adolescents with Anorexia Nervosa: Length-of-Stay Predictors, Cardiovascular Recovery, Refeeding Safety, and Psychiatric Comorbidity Burden

**DOI:** 10.3390/nu18172789

**Published:** 2026-08-26

**Authors:** Saba F. Elhag, Abdulla Fahmi, Abdulrahman Alansari, Sara Al-Kuwari, Amal Saleh Al-Kuwari, Amna Ahmed, Shahd Hani Jaouni, Amine Zaidi, Tasnim L. Milhem, Reem Bachir, Moayad H. Ali, Asma Ahmed, Qusi Shaban, Sheema Hashem, Madeeha Kamal

**Affiliations:** 1 Department of Human Genetics, Sidra Medicine, Al Gharrafa Street, Al Rayyan, Doha 26999, Qatar; selmubarakelhag@sidra.org (S.F.E.); shashem@sidra.org (S.H.); 2 College of Health and Life Sciences, Hamad Bin Khalifa University (HBKU), Doha 26999, Qatar; 3 Department of Adolescent Medicine, Sidra Medicine, Al Gharrafa Street, Al Rayyan, Doha 26999, Qatar; afahmi@sidra.org; 4 Department of Family Medicine, Hamad Medical Corporation, Doha P.O. Box 3050, Qatar; alansari10@hamad.qa; 5 Department of Pediatrics, Sidra Medicine, Al Gharrafa Street, Al Rayyan, Doha 26999, Qatar; salkuwari4@sidra.org (S.A.-K.); aalkuwari7@sidra.org (A.S.A.-K.); aahmed18@sidra.org (A.A.); sjaouni1@sidra.org (S.H.J.); azaidi2@sidra.org (A.Z.); tmilhem@sidra.org (T.L.M.); rbachir@sidra.org (R.B.); aahmed29@sidra.org (A.A.); qshaban@sidra.org (Q.S.); 6 Department of Pediatrics, Hamad Medical Corporation, Doha P.O. Box 3050, Qatar; v-mabdelhameed@hamad.qa; 7 Weill Cornell Medical College–Qatar (WCM-Q), Cornell University, Al Gharrafa Street, Al Rayyan, Doha 26999, Qatar; 8 College of Medicine, QU Health, Qatar University, Al Gharrafa Street, Al Rayyan, Doha 26999, Qatar

**Keywords:** anorexia nervosa, adolescents, medical stabilization, bradycardia, length of stay, BMI z-score, nutritional rehabilitation, refeeding syndrome, psychiatric comorbidity, family-based therapy

## Abstract

**Background/Objectives:** Adolescents with anorexia nervosa (AN) face substantial medical and psychiatric risk during hospitalization. We examined predictors of length of stay (LOS), cardiovascular recovery, refeeding safety, and psychiatric comorbidity in a consecutive pediatric cohort at a national tertiary center. **Methods:** Retrospective cohort of 68 consecutively admitted adolescents (91.2% female; mean age 14.2 ± 1.8 years, range 8–17; 85.3% restricting type) admitted between January 2017 and May 2025. A structured nutritional rehabilitation protocol (1000–1200 kcal/day, escalating by 200 kcal/day every 48 h to 2500–3000 kcal/day) was implemented with daily serum electrolyte monitoring. Statistical analyses included paired tests, Spearman correlations, multivariable regression of log-transformed LOS, and partial correlations controlling for the admission BMI z-score. **Results:** Nutritional and cardiovascular parameters improved from admission to discharge. Mean supine heart rate (HR) increased by 28.4 bpm (95% CI 25.0–31.8; *p* < 0.001), and bradycardia prevalence (HR < 50 bpm) improved from 39.7% to 1.5%. Lower admission BMI z-score was associated with longer LOS; each 1-unit higher BMI z-score was associated with an 18.9% shorter LOS in adjusted log-LOS regression (*p* = 0.003). Exploratory analyses of HR recovery suggested that the magnitude of HR improvement was more closely associated with baseline BMI z-score and baseline supine HR than with caloric escalation. Systematic electrolyte monitoring documented no hypophosphatemia requiring intervention and no refeeding syndrome. Documented mental health comorbidities or clinically significant symptom clusters were present in 82.4% of patients. **Conclusions:** In this adolescent anorexia nervosa cohort, structured inpatient nutritional rehabilitation was associated with substantial cardiovascular and nutritional improvement. Baseline nutritional status was associated with hospital course and cardiovascular response, while LOS should be interpreted as a composite outcome influenced by nutritional severity, discharge criteria, mental health needs, and family readiness. The absence of refeeding complications is reassuring and suggests that the protocol was metabolically safe under systematic electrolyte monitoring in this specialized tertiary setting.

## 1. Introduction

Anorexia nervosa (AN) carries one of the highest mortality rates among psychiatric disorders, with a standardized mortality ratio of approximately 5.9 reported in a meta-analysis [1]. The peak onset occurs between 13 and 18 years, and the consequences of prolonged malnutrition during this critical developmental window are particularly severe [1,2,3]. Bradycardia, defined as a resting supine heart rate (HR) below 50 bpm, is the most frequently reported indication for inpatient medical admission in adolescents with AN, and has been documented in up to 80% of patients in hospitalized pediatric series [4,5]. Additional cardiovascular manifestations include orthostatic hypotension, which contributes to the risk of hemodynamic compromise and sudden cardiac death [6].

Medical stabilization requires careful nutritional rehabilitation alongside continuous cardiovascular monitoring. Observational studies and systematic reviews have shown that higher caloric intake is associated with faster weight restoration and shorter hospital stays without increasing rates of electrolyte complications [7,8,9]. The Society for Adolescent Health and Medicine (SAHM) position statement and contemporary guideline evidence increasingly support protocolized, medically monitored nutritional rehabilitation that avoids both undernutrition and refeeding syndrome, with specialist multidisciplinary oversight [10,11]. However, the mechanism by which nutritional restitution restores cardiovascular function remains incompletely characterized. A critical methodological challenge is confounding by baseline nutritional status: more severely malnourished patients present with both lower resting HR and lower caloric intake as co-manifestations of the same disease process, making it essential to disentangle the independent contributions of nutritional status and caloric escalation to cardiovascular recovery [12].

Refeeding syndrome, characterized by acute hypophosphatemia and potentially fatal electrolyte shifts during nutritional rehabilitation, has historically led to conservative ‘start low, go slow’ caloric approaches [13,14]. Accumulating pediatric evidence, including the multicenter STRONG randomized trial [15] suggests that refeeding syndrome is less common than previously feared in adolescents with AN managed in specialized settings with systematic electrolyte monitoring, and that the risks of underfeeding may outweigh those of refeeding [9,15]. Systematic documentation of electrolyte profiles during aggressive nutritional rehabilitation provides essential safety data to inform contemporary clinical practice.

Beyond the medical domain, mental health comorbidity in AN is substantial and clinically significant. Depression, anxiety disorders, suicidal ideation, and self-harm behaviors co-occur with AN at high rates and influence treatment engagement, the hospital course, and long-term outcomes [16,17]. Despite their clinical importance, psychiatric comorbidities are systematically undercharacterized in medical stabilization cohorts, where the primary focus has naturally been physiological recovery. Furthermore, the transition from inpatient medical stabilization to outpatient care is a high-risk period for relapse, and family-based therapy (FBT), in which parents are empowered as the primary agents of nutritional restoration, is the best-evidenced psychotherapeutic approach for adolescents with AN [18,19].

The present study addresses these gaps through a retrospective analysis of 68 consecutively admitted adolescents with AN at a national tertiary pediatric center, using a structured, protocol-driven nutritional rehabilitation approach. Specific aims were to: (1) characterize clinical and nutritional changes during inpatient medical stabilization; (2) identify predictors of LOS using multivariable regression; (3) explore associations between caloric rehabilitation and HR recovery using partial correlations to account for baseline nutritional status, recognizing that this approach reduces but does not remove confounding by indication; (4) assess electrolyte safety during aggressive refeeding; (5) describe the prevalence and pattern of psychiatric comorbidities; and (6) describe the discharge criteria and structured outpatient follow-up pathway of Sidra Medicine, the national tertiary pediatric referral center for eating disorders in Qatar and the sole dedicated inpatient eating disorders medical stabilization program.

## 2. Materials and Methods

### 2.1. Study Design, Setting, and Participants

This retrospective observational cohort study was conducted at a national tertiary pediatric center with a dedicated inpatient program for eating disorders. Participants were adolescents aged 8–17 years who were consecutively admitted for medical stabilization of AN between January 2017 and May 2025. Inclusion criteria were: (1) a confirmed DSM-5-TR diagnosis of AN (restricting or binge-purge subtype) established by the treating physician [20]; (2) inpatient admission for medical stabilization with complete admission and discharge clinical data; and (3) age ≤ 18 years at admission. Patients were excluded if they had an alternative primary diagnosis, incomplete admission records, or a hospital stay of fewer than 24 h. Three patients (4.4%) had a documented prior hospitalization for AN-related medical instability within five years preceding the index admission.

The study was conducted in accordance with the Declaration of Helsinki and approved by the Institutional Review Board of Sidra Medicine (IRB no: 1929504), which waived the requirement for individual informed consent given the retrospective, de-identified nature of the data. This study is reported in accordance with the Strengthening the Reporting of Observational Studies in Epidemiology (STROBE) guideline [21]; a completed STROBE checklist is provided as Supplementary Material S1.

### 2.2. Clinical Variables and Outcome Measures

The following variables were extracted from electronic medical records at admission and discharge: body weight (kg), height (cm), body mass index (BMI, kg/m^2^), BMI z-score (age- and sex-standardized using WHO reference standards), BMI-for-age percentile (BMI%ile, range 0–100th centile, derived from the age- and sex-specific BMI z-score using WHO growth references; recorded as ‘%mBMI’ in the source dataset; this variable does not represent the percent-median-BMI formula [patient BMI ÷ 50th-percentile BMI × 100]), resting supine HR (bpm), standing HR (bpm), systolic blood pressure (SBP, mmHg), and diastolic blood pressure (DBP, mmHg). Prescribed daily caloric intake at admission and discharge (kcal/day) was obtained from dietitian records. Serum electrolytes obtained within 24 h of admission included sodium, potassium, chloride, blood urea nitrogen, creatinine, phosphate, calcium, magnesium, and bicarbonate. Mental health comorbidities were extracted from treating psychiatrist documentation and coded as binary variables. LOS was defined as the number of days between admission and discharge. Medical instability was assessed using four binary criteria: (1) HR < 50 bpm; (2) SBP < 90 mmHg; (3) BMI < 15 kg/m^2^; and (4) BMI z-score < −2.

### 2.3. Inpatient Nutritional Rehabilitation Protocol

All patients were admitted to a supervised medical ward with continuous cardiac telemetry. Activity was restricted to bed rest, with graduated mobilization as cardiovascular parameters normalized. The nutritional rehabilitation protocol was guided by the American Academy of Pediatrics and SAHM clinical practice guidelines for pediatric eating disorders [5,11].

The initial caloric prescription was determined by the treating physician. Patients consuming ≤500 kcal/day before admission were commenced at 1000 kcal/day, whereas those consuming >500 kcal/day were commenced at 1200 kcal/day. Calories were advanced by 200 kcal/day every 48 h, with adjustments at the treating team’s discretion based on weight-gain trajectory, electrolyte results, and individual tolerance. Target discharge intake was 2500–3000 kcal/day, divided into three meals and two to three snacks. All meals were supervised by nursing staff. If a patient was unable to complete a meal within 45 min, the shortfall was supplemented with a high-calorie oral nutritional supplement (Pediasure^®^ 1.5 kcal/mL). Nasogastric tube feeding was initiated in 1–2 patients (approximately 2–3% of the cohort) and used for 1–2 days before oral intake was resumed.

Serum electrolytes, including phosphate and magnesium, were measured daily from day 2 through day 7 of admission. If all values remained within normal limits after this initial monitoring period, frequency was reduced to every 48–72 h or as clinically indicated. Hypophosphatemia (phosphate < 0.81 mmol/L) or hypomagnesemia (magnesium < 0.70 mmol/L) prompted electrolyte replacement and continuation of daily monitoring until normalization. Prophylactic phosphate supplementation was not administered to patients with normal electrolyte levels on admission. A standard micronutrient supplement was prescribed throughout each admission. Daily weight, supine and standing HR, and blood pressure were monitored throughout the stay.

### 2.4. Discharge Criteria

Medical discharge was considered when patients met all of the following criteria: (1) sustained cardiovascular normalization (resting supine HR ≥ 60 bpm; absence of orthostatic HR rise > 35 bpm from supine to standing; SBP ≥ 90 mmHg) on at least two consecutive days; (2) reliable completion of three meals and two snacks orally; (3) successful completion of at least one supervised home meal, a family-prepared meal eaten under nurse supervision in the inpatient setting, consistent with FBT principles [18]; (4) ability to eat a family meal at home without significant difficulty; and (5) multidisciplinary team discharge assessment involving the pediatrician, child psychiatrist, clinical psychologist, dietitian, social worker, and clinical nurse specialist.

Following medical discharge, all patients were enrolled in a structured multidisciplinary outpatient program. Weekly clinic reviews initially involved the pediatrician, dietitian, and clinical psychologist, with measurement of weight, HR, and blood pressure at each visit. Once regular eating was established without major difficulty, review frequency was reduced to monthly. Parental competency in FBT-based home nutritional management was reinforced throughout. Total outpatient follow-up was typically 1 to 2 years, with step-down to primary care upon the achievement of recovery goals.

### 2.5. Statistical Analysis

Continuous variables are presented as mean ± standard deviation (SD) and median [interquartile range, IQR]; categorical variables as frequency and percentage. Change from admission to discharge was assessed using a paired Student’s *t*-test (normally distributed variables) and a Wilcoxon signed-rank test (non-normal or skewed variables). McNemar’s test was used to compare the proportions meeting each medical instability criterion at admission and at discharge.

Spearman rank correlations were used to assess associations between potential predictors and LOS. LOS was log-transformed (natural logarithm) prior to multivariable linear regression to address its strong right skewness. Multivariable models included age, sex, and AN subtype as covariates, along with the primary nutritional predictor. A negative binomial regression model was fitted as a complementary approach. Bivariate and partial Pearson correlations were computed for caloric–HR relationships; partial correlations controlled for admission BMI z-score using the ppcor R package. Multivariable linear regression with HR improvement as the outcome identified independent predictors. Two pre-specified sensitivity analyses were performed: (A) excluding the single patient with LOS > 180 days; (B) excluding the single patient with admission caloric intake < 100 kcal/day. All analyses were conducted in R version 4.3 (R Core Team, Vienna, Austria). Given the exploratory nature of multiple comparisons, all *p*-values are unadjusted, and results should be interpreted accordingly. Statistical significance was set at α = 0.05 (two-tailed).

## 3. Results

### 3.1. Baseline Characteristics

Sixty-eight adolescents (62 female, 91.2%; 6 male, 8.8%) were included. Mean age at admission was 14.2 ± 1.8 years (range 8–17). AN restricting type (AN-R) was diagnosed in 58 patients (85.3%) and AN binge-purge type (AN-BP) in 10 (14.7%). The majority (53/68, 77.9%) were admitted primarily for bradycardia. Baseline characteristics are presented in Table 1.

**Figure 1:**
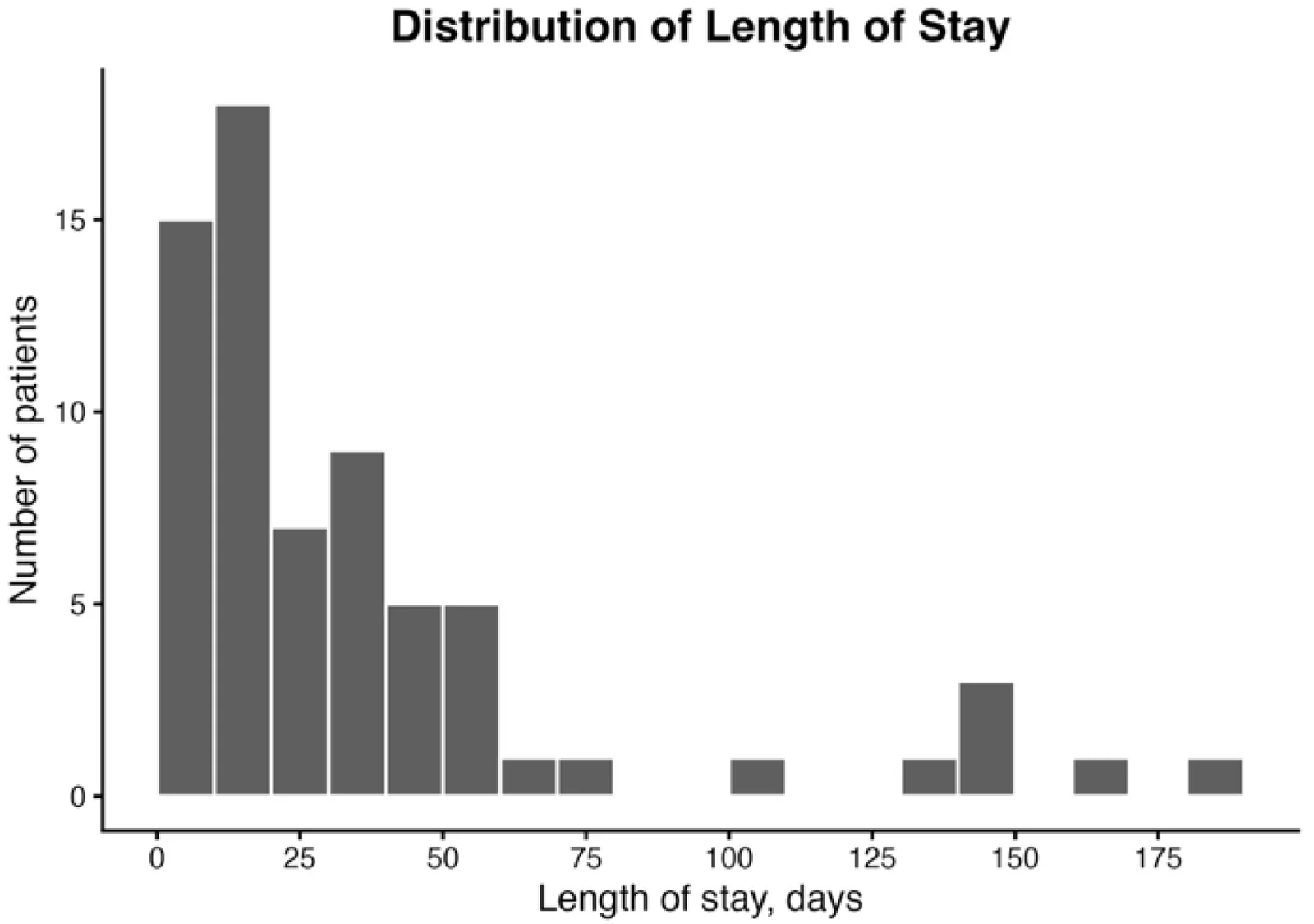
Distribution of length of stay (LOS) in days for the 68 hospitalized adolescents with anorexia nervosa (AN). The distribution is strongly right-skewed (median 24 days, IQR 11–41; mean 36.9 ± 41.6 days). One patient had a prolonged inpatient stay of 188 days; this admission was driven by severe psychiatric comorbidities requiring extended psychiatric stabilization before discharge could be safely arranged, rather than by persistent nutritional or cardiovascular failure.

Patients were severely malnourished at admission: mean BMI z-score was −2.16 ± 1.85 and mean BMI was 16.06 ± 2.75 kg/m^2^. Mean resting supine HR was 52.9 ± 10.8 bpm and mean SBP was 91.6 ± 7.4 mmHg. Mean admission caloric intake was 1231 ± 383 kcal/day. Median LOS was 24 days [IQR 11–41], with a mean of 36.9 ± 41.6 days; the distribution was strongly right-skewed (Figure 1). AN-R and AN-BP subtypes were well matched on all baseline parameters except diastolic BP (61.2 vs. 56.2 mmHg, *p* = 0.023). Given the small AN-BP sample (*n* = 10), all subtype comparisons should be interpreted with caution. Admission electrolyte values were largely within normal reference ranges for both subtypes.

### 3.2. Nutritional and Clinical Changes During Admission

**Figure 2:**
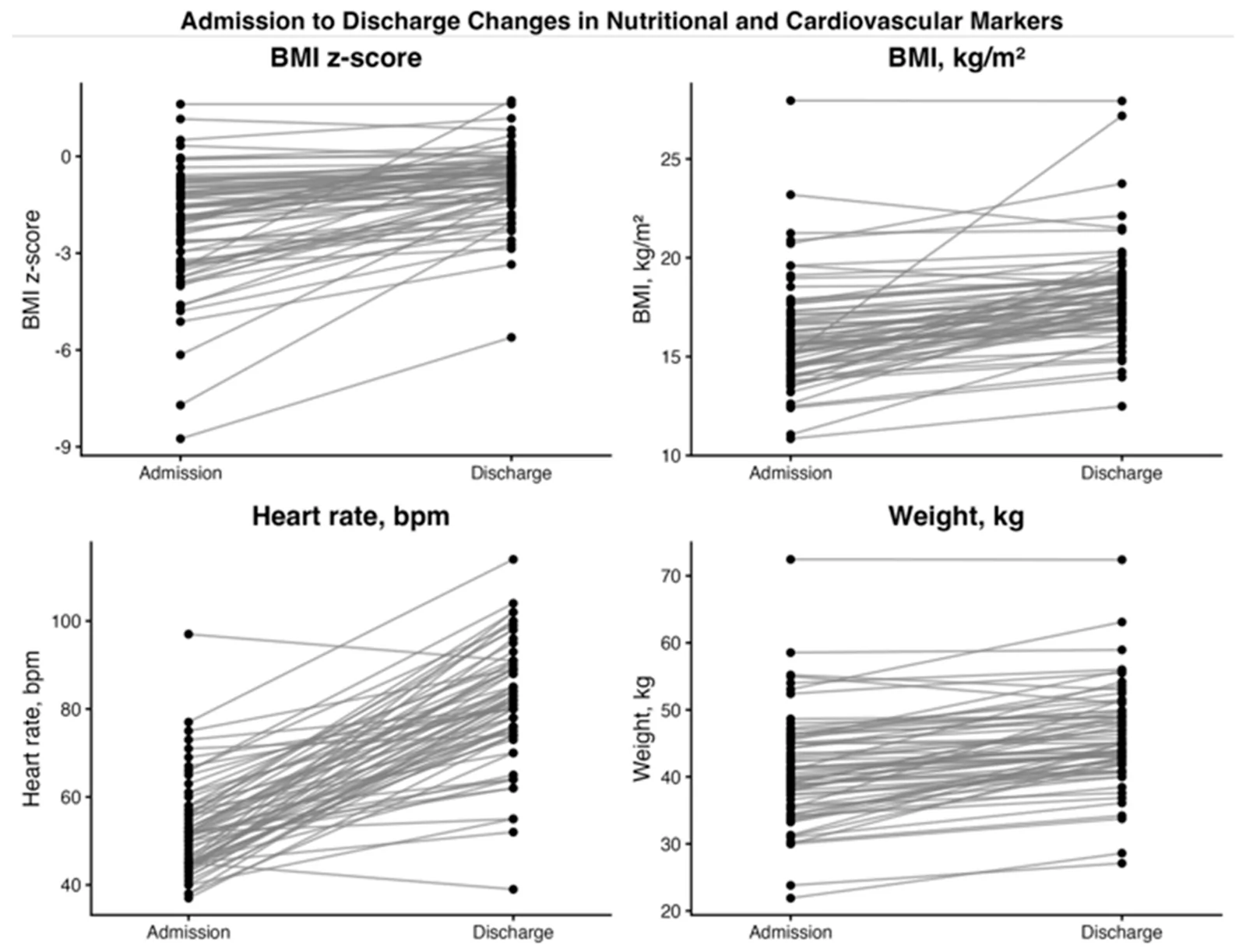
Individual patient trajectories from admission to discharge for BMI z-score, BMI (kg/m^2^), heart rate (bpm), and body weight (kg). Each line represents one patient. The broad spread of slopes reflects substantial inter-individual heterogeneity in the severity of malnutrition and cardiovascular compromise at presentation, and in the degree of recovery achieved during the inpatient stay.

All monitored parameters improved from admission to discharge (Table 2; Figure 2). The largest changes were in supine heart rate, which rose by a mean of 28.4 bpm (95% CI 25.0–31.8), and in prescribed caloric intake, which rose by 1442 kcal/day (95% CI 1242–1642). Weight, BMI, BMI z-score and both blood pressures also improved significantly on paired testing; the individual estimates are given in Table 2 and are not repeated here. All comparisons reached *p* < 0.001.

### 3.3. Resolution of Medical Instability

**Figure 3:**
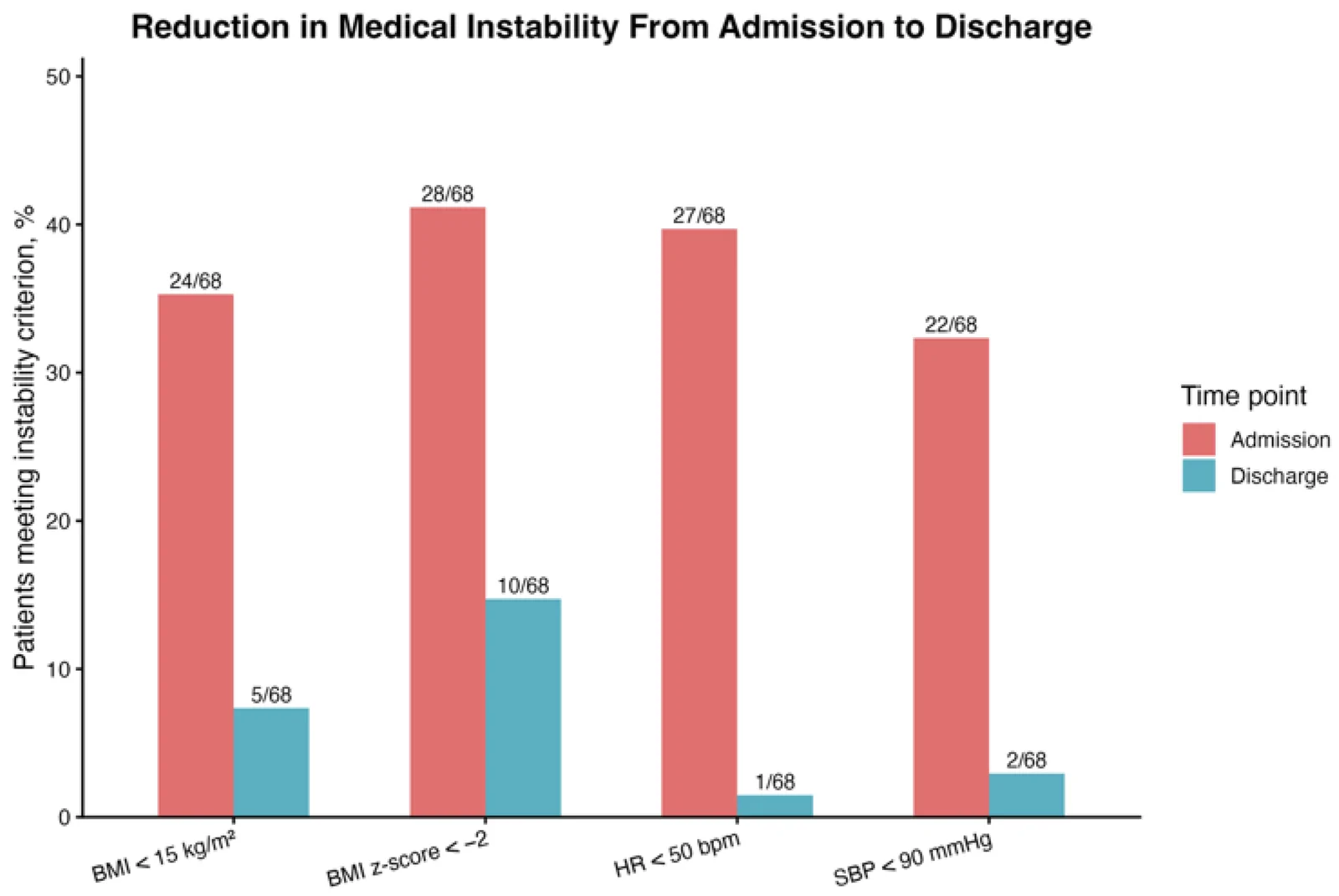
Reduction in medical instability criteria from admission (red) to discharge (teal) in 68 hospitalized adolescents with anorexia nervosa. Proportions are displayed for each of the four pre-specified criteria: bradycardia (HR < 50 bpm), hypotension (SBP < 90 mmHg), severe underweight (BMI < 15 kg/m^2^), and significant nutritional compromise (BMI z-score < −2). Numbers above bars indicate absolute counts. All criteria improved significantly by discharge using McNemar’s test. The underlying numerical data are presented in Table 3.

Each of the four pre-specified medical instability criteria showed highly significant improvement from admission to discharge (Table 3; Figure 3). Bradycardia (HR < 50 bpm) was present in 27/68 patients (39.7%) at admission and resolved to 1/68 (1.5%) by discharge (McNemar *p* < 0.001). Hypotension (SBP < 90 mmHg) resolved in 21 out of 22 affected patients (32.4% to 2.9%; *p* < 0.001). The proportion with BMI < 15 kg/m^2^ fell from 35.3% to 7.4%, and the proportion with BMI z-score < −2 fell from 41.2% to 14.7% (both *p* < 0.001). No patient worsened on any criterion.

### 3.4. Predictors of Length of Stay

Spearman correlations of all assessed predictors with LOS are presented in Table 4. The strongest correlates were measures of nutritional improvement achieved during admission: weight change (ρ = 0.682, *p* < 0.001), BMI z-score change (ρ = 0.656, *p* < 0.001), and caloric increase (ρ = 0.427, *p* < 0.001)—reflecting that patients who remained hospitalized longer achieved greater nutritional restitution (Supplementary Figure S3). Among the admission-time variables, lower BMI z-score (ρ = −0.472, *p* < 0.001) and lower BMI%ile (ρ = −0.467, *p* < 0.001) were most strongly associated with longer LOS (Supplementary Figures S1 and S2).

**Figure 4:**
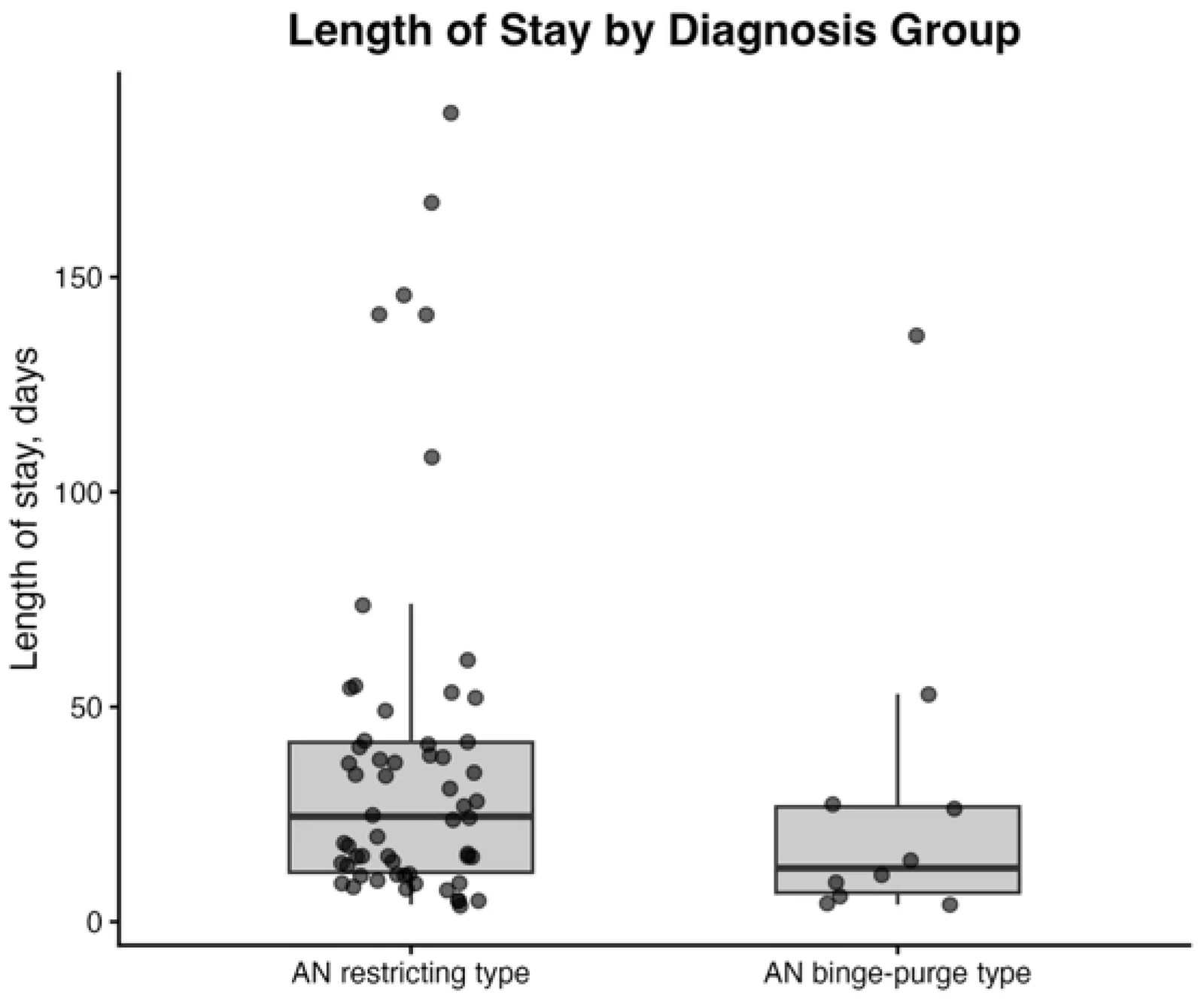
Length of stay (LOS) in days by AN diagnostic subtype. Box plots show median and interquartile range; individual patient data points are overlaid. LOS did not differ significantly between AN restricting type (AN-R, *n* = 58) and AN binge-purge type (AN-BP, *n* = 10) by Wilcoxon rank-sum test (*p* > 0.05). The AN-BP subgroup was small, and this comparison should be interpreted with caution.

In log-LOS multivariable regression adjusted for age, sex, and AN subtype, each 1-unit increment in admission BMI z-score was associated with an 18.9% shorter LOS (β = −0.209, 95% CI −0.340 to −0.076; *p* = 0.003; model R^2^ = 0.167). Each 1 percentile-point increment in admission BMI%ile was associated with a 1.7% shorter LOS (β = −0.018, 95% CI −0.028 to −0.007; *p* = 0.002; R^2^ = 0.170). The negative binomial model confirmed the primary result: each 1-unit increment in BMI z-score was associated with a 17.5% reduction in expected LOS (rate ratio 0.825, 95% CI 0.711–0.950; *p* = 0.002). Results were unchanged in sensitivity analysis A (excluding the LOS = 188-day outlier; β = −0.216, *p* = 0.001). The admission bradycardia indication was not an independent predictor of LOS in either model (*p* > 0.60). LOS did not differ significantly between AN-R and AN-BP subtypes (Figure 4; Wilcoxon *p* > 0.05), though the AN-BP subgroup was small (*n* = 10). These findings are consistent with prior pediatric cohort data identifying lower nutritional status at admission as the primary driver of hospitalization duration [22].

### 3.5. Caloric Rehabilitation and Cardiovascular Recovery

Bivariate and partial correlations between caloric intake and HR parameters are presented in Table 5. Two qualitatively distinct patterns were identified.

First, admission caloric intake was positively correlated with admission supine HR (Pearson r = 0.324, *p* = 0.007; Supplementary Figure S6). This association persisted after partial correlation controlling for admission BMI z-score (partial r = 0.297, *p* = 0.014), indicating that the calorie–HR cross-sectional association reflects shared disease severity rather than a simple nutritional-status confound. In the sensitivity analysis excluding the patient with admission calories < 100 kcal/day, this association strengthened (r = 0.462, *p* < 0.001; partial r = 0.437, *p* < 0.001).

**Figure 5:**
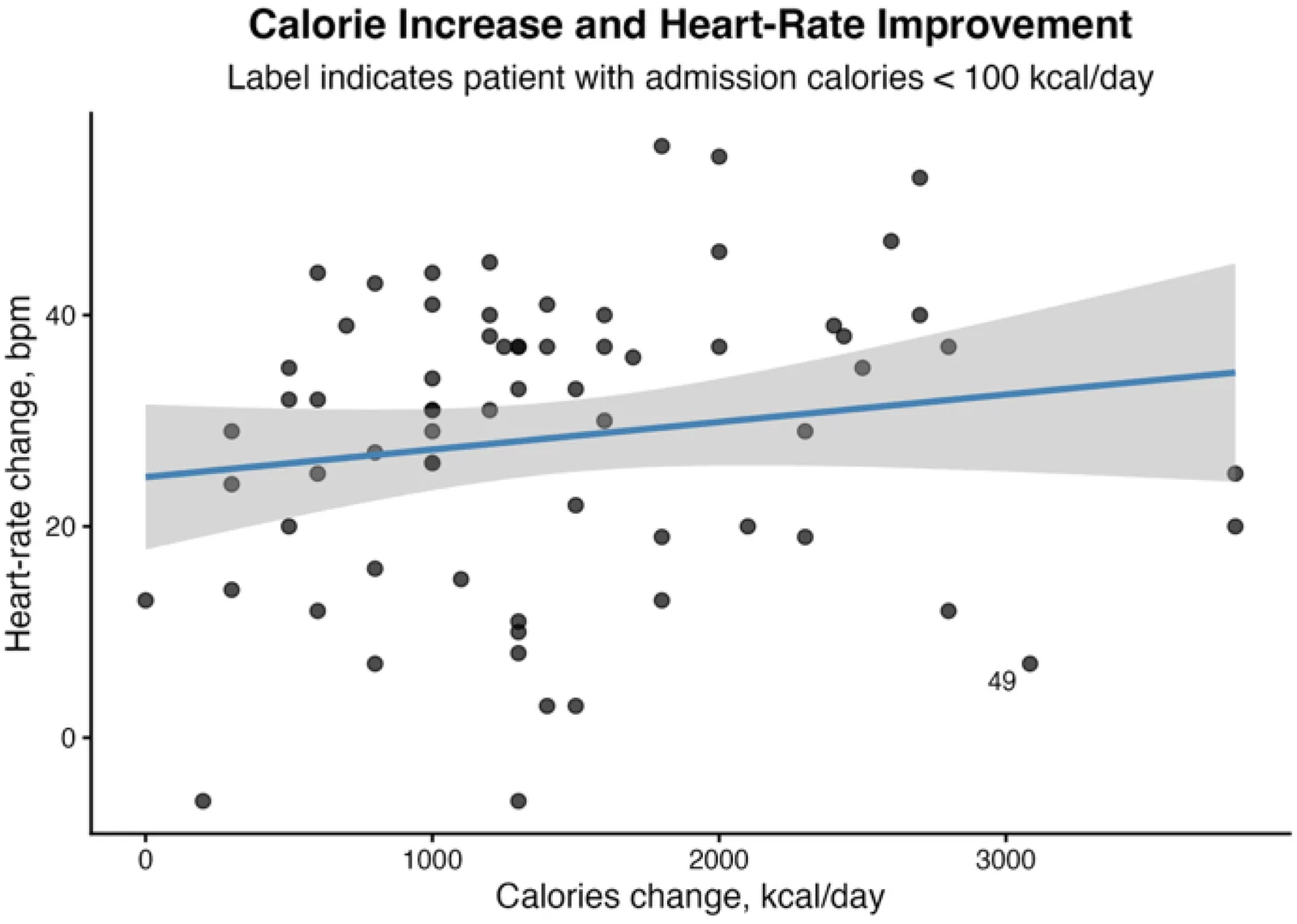
Calorie increase from admission to discharge and heart-rate improvement (bpm) in 68 adolescents with anorexia nervosa. Each dot represents one patient. The regression line (blue) and 95% confidence band (grey) showed no significant association (Pearson r = 0.153, *p* = 0.214; partial r controlling for admission BMI z-score = 0.052, *p* = 0.674). The label indicates the patient with admission calories < 100 kcal/day, who was examined in a pre-specified sensitivity analysis (Supplementary Figure S4).

Second, although heart-rate recovery occurred in parallel with nutritional rehabilitation, the magnitude of HR improvement was not primarily explained by caloric escalation alone. The unadjusted association between caloric increase and HR improvement was weak and not statistically significant (Pearson r = 0.153, *p* = 0.214; Figure 5). After controlling for admission BMI z-score, caloric escalation showed no additional independent association with HR improvement (partial r = 0.052, *p* = 0.674). This finding was unchanged after exclusion of the low-admission-calorie outlier (Supplementary Figure S4; partial r = 0.108, *p* = 0.387), and discharge caloric intake was not significantly associated with discharge HR (Supplementary Figure S5; Pearson r = 0.150, *p* = 0.222). Together, these results suggest that cardiovascular recovery during admission was more closely related to baseline nutritional and cardiovascular severity and overall nutritional restoration than to the absolute degree of caloric escalation.

### 3.6. Multivariable Predictors of Heart-Rate Improvement

In multivariable linear regression with HR improvement as the outcome (model R^2^ = 0.319, adjusted R^2^ = 0.252; Table 6), caloric change per 1000 kcal was not a significant predictor (β = −0.48, 95% CI −4.48 to +3.52; *p* = 0.812). Three independent predictors were identified: (1) admission BMI z-score (β = −2.70, 95% CI −4.65 to −0.74; *p* = 0.008)—more severe malnutrition at admission was associated with greater HR recovery; (2) admission supine HR (β = −0.476, 95% CI −0.767 to −0.186; *p* = 0.002)—a regression-to-the-mean effect whereby patients with more marked bradycardia showed greater absolute improvement; and (3) age at admission (β = −2.13, 95% CI −3.97 to −0.29; *p* = 0.024)—younger patients showed greater HR improvement. Sex and AN subtype were not significantly associated with HR improvement in the multivariable model.

### 3.7. Electrolyte Profile and Refeeding Safety

Admission electrolyte values were available for all 68 patients. Mean phosphate was 1.30 ± 0.16 mmol/L (reference range 0.81–1.45 mmol/L). No patient met the threshold for hypophosphatemia (phosphate < 0.81 mmol/L) or severe hypophosphatemia (<0.60 mmol/L) at admission. No case of refeeding syndrome was documented during any admission. On a protocol of systematic daily electrolyte monitoring from day 2 through day 7, no patient developed hypophosphatemia requiring intervention during the refeeding period.

Other electrolyte abnormalities at admission were uncommon. Hypokalemia (potassium < 3.5 mmol/L) was the most frequent, present in 5/68 patients (7.4%). Metabolic acidosis (bicarbonate < 22 mmol/L) was present in 4/68 (5.9%) and metabolic alkalosis (bicarbonate > 29 mmol/L) in 2/68 (2.9%). Hyponatremia, hypochloremia, hypocalcemia, and hypomagnesemia were each present in ≤3% of patients. No significant differences in electrolyte profiles were found between AN-R and AN-BP subtypes (all *p* > 0.10).

### 3.8. Medical and Mental Health Comorbidities

At least one concurrent medical or mental health diagnosis was documented in 56/68 patients (82.4%; Table 7). Depression was the most prevalent comorbidity (58.8%), followed by anxiety disorder (42.6%), amenorrhea (27.9%), suicidal ideation or attempt (19.1%), and self-harm behavior (17.6%). No statistically significant differences in comorbidity prevalence were found between AN-R and AN-BP subtypes for any category. Given the small AN-BP sample (*n* = 10), these subtype comparisons should be interpreted with caution. Given the high overall comorbidity prevalence, the subgroup without any documented psychiatric comorbidity was small (*n* = 12, 17.6%). Formal subgroup comparisons of LOS or HR improvement by comorbidity status were not performed, as the minimum detectable differences substantially exceeded any plausible clinically meaningful threshold.

## 4. Discussion

This retrospective cohort describes inpatient nutritional rehabilitation and medical stabilization among 68 consecutively hospitalized adolescents with AN in a tertiary pediatric eating-disorders program. Five main observations emerged. First, structured inpatient nutritional rehabilitation was associated with clinically meaningful improvement in nutritional, cardiovascular, and hemodynamic parameters. Second, lower admission BMI z-score was associated with longer LOS. Third, HR recovery occurred alongside nutritional rehabilitation, but exploratory adjusted analyses suggested that the magnitude of HR improvement was more closely associated with baseline nutritional and cardiovascular severity than with caloric escalation alone. Fourth, systematic electrolyte monitoring documented no hypophosphatemia requiring intervention and no refeeding syndrome. Fifth, documented mental health comorbidities or clinically significant symptom clusters were common. These findings should be interpreted as observational and hypothesis-generating rather than causal.

The cardiovascular improvements in this cohort were clinically dramatic. A mean HR increase of 28.4 bpm, representing a 54% increase from the mean admission HR of 52.9 bpm, together with near-complete resolution of bradycardia (39.7% to 1.5%), indicates clinically meaningful cardiovascular improvement during protocol-driven nutritional rehabilitation, consistent with prior pediatric studies [4,6]. However, because this study was retrospective and uncontrolled, these findings should not be interpreted as establishing treatment efficacy. Individual patient trajectories (Figure 2) revealed substantial heterogeneity, underscoring that the presentation of AN and its cardiovascular manifestations vary widely even within a clinically defined cohort. Concurrent improvements in blood pressure and weight reflect global hemodynamic and metabolic recovery associated with nutritional restitution.

Lower admission BMI z-score was associated with longer LOS, which is clinically plausible and consistent with prior pediatric cohort data showing an association between lower nutritional status at admission and longer hospital stay [22]. In the present cohort, each 1-unit increase in admission BMI z-score was associated with an 18.9% shorter LOS, corresponding to approximately 4.5 fewer inpatient days at the observed median stay of 24 days. This effect size is clinically meaningful, but LOS should not be interpreted as a direct biological marker of illness severity or nutritional recovery alone. In this program, discharge required cardiovascular stabilization, attainment of the caloric threshold, successful completion of a supervised family meal within the hospital, and multidisciplinary agreement on psychosocial readiness for discharge. Therefore, LOS reflects the combined influence of baseline nutritional severity, clinical recovery, mental health needs, institutional discharge policy, and family readiness. These findings support early recognition and timely referral before severe malnutrition develops, but implications for hospital resource use require evaluation in prospective studies and across settings with different admission and discharge criteria.

The relationship between caloric rehabilitation and cardiovascular recovery requires careful interpretation. The significant positive correlation between admission caloric intake and admission HR (r = 0.324, *p* = 0.007), which persisted after controlling for BMI z-score (partial r = 0.297, *p* = 0.014; Supplementary Figure S6), reflects a cross-sectional severity signal: patients consuming more calories at admission had better nutritional status and higher resting HRs, both co-manifestations of less severe disease. This is not evidence of a causal benefit of caloric intake on HR; the treating clinician’s caloric prescription is itself informed by clinical severity, introducing a confounding-by-indication pathway that partial correlations controlling for BMI z-score alone cannot fully eliminate. In contrast, the change in caloric intake from admission to discharge was unrelated to the degree of HR improvement across multiple analytical approaches (partial r = 0.052, *p* = 0.674; Figure 5; sensitivity analysis, Supplementary Figure S4). Multivariable regression confirmed that HR recovery was independently driven by baseline BMI z-score (β = −2.70, *p* = 0.008) and by a regression-to-the-mean effect of admission HR, not by caloric escalation. These results indicate that caloric escalation added no independent predictive value for heart-rate recovery once baseline severity was accounted for. This is not the same as showing that caloric intake is unimportant. Cardiovascular recovery in AN reflects several processes we did not measure, including autonomic reconditioning, restoration of fat-free mass, rehydration, graded reduction in activity restriction and improved sleep, and the present sample was powered to detect only moderate effects. Aggressive caloric escalation remains clinically necessary for efficient weight restoration; what these data suggest is that its cardiovascular benefit is not separable from the nutritional recovery it produces.

The refeeding safety profile of this cohort builds on the emerging literature challenging historical refeeding conservatism. No cases of hypophosphatemia occurred among the 68 patients managed within this specialized tertiary setting with a protocol that started at 1000–1200 kcal/day. This aligns with randomized trial evidence and large observational cohorts showing that clinically significant hypophosphatemia during refeeding is far less common than previously feared in adolescents without severe pre-existing electrolyte abnormalities [9,15,23]. Crucially, this safety record was achieved within a framework of systematic daily electrolyte monitoring from day 2 through day 7: the absence of any clinically significant refeeding syndrome episode supports the adequacy of the protocol, though retrospective data alone cannot determine whether safety reflected low intrinsic risk or monitoring-enabled timely detection. The current results should not be interpreted as evidence that aggressive refeeding is universally safe outside of specialized settings with comparable monitoring protocols. Hypokalemia (7.4% at admission) and metabolic acidosis (5.9%) were the most common electrolyte disturbances, consistent with the physiological consequences of prolonged caloric restriction and in the AN-BP subgroup, purging behavior [24].

The prevalence of psychiatric comorbidity in this cohort, 82.4% overall, with depression in 58.8% and anxiety in 42.6%, confirms and extends findings from the broader AN literature [16,17] and has direct implications for the structure of inpatient care. Crucially, suicidal ideation or attempt (19.1%) and self-harm behavior (17.6%) were identified in a medically admitted cohort: populations often assumed to be at the less severe end of the psychiatric complexity spectrum because they present to pediatric medicine rather than to psychiatry. One patient had a prolonged inpatient stay driven entirely by psychiatric stabilization barriers rather than nutritional or cardiovascular failure, highlighting the extent to which psychiatric complexity can dominate the clinical trajectory even within a medical stabilization program. The comorbidity burden observed here is consistent with a model of care in which child psychiatry, clinical psychology, and social work are involved from the day of admission rather than as secondary consultants. We note that the present design did not compare staffing models and therefore cannot demonstrate that such a structure improves outcomes; this is offered as a clinical implication of the observed comorbidity prevalence rather than as a finding of the study.

The discharge protocol used at this center operationalizes FBT principles during the inpatient-to-outpatient transition [18,19]. Discharge was contingent not only on cardiovascular normalization and attainment of a 2500–3000 kcal/day oral intake threshold, but also on the successful completion of at least one supervised family meal before leaving the hospital, explicitly empowering parents as the primary agents of nutritional restoration before discharge, consistent with the central mechanism of FBT for adolescent AN. Although the current retrospective design did not capture post-discharge weight trajectory or readmission rates as structured outcomes, the three prior hospitalizations identified in this cohort underscore the ongoing relapse risk and the need for sustained, coordinated outpatient support. Prospective evaluation of FBT-informed discharge criteria on readmission rates is an important direction for future research.

### Strengths and Limitations

The principal strengths of this study are methodological and contextual. The consecutive cohort design over eight years, together with complete primary inpatient outcome data, reduces but does not eliminate selection bias and provides clinically useful information from a national tertiary referral program. The analytic approach, including adjusted models and partial correlations, allowed exploratory assessment of the relationship between baseline nutritional severity, caloric rehabilitation, LOS, and HR recovery; however, these methods reduce rather than remove confounding, particularly because clinicians determined caloric prescriptions and adjusted them during admission. The absence of hypophosphatemia requiring intervention or documented refeeding syndrome provides reassuring safety data within a closely monitored tertiary inpatient setting. The explicit description of the nutritional rehabilitation protocol, discharge criteria, and outpatient pathway enables comparison with other centers.

Five limitations warrant discussion. First, this is a single-center study; however, as the national tertiary referral center for pediatric eating disorders, the consecutive design over eight years captures the complete national inpatient experience for this condition during that period. Second, the absence of post-discharge outcome data, weight trajectory, readmission rates, and eating disorder psychopathology is the most clinically meaningful gap. Inpatient medical stabilization is only the first step of recovery, and without longitudinal follow-up, we cannot evaluate whether the discharge protocol and FBT-based handover translate into durable improvements. Third, psychiatric comorbidities were ascertained from clinical documentation rather than from structured research interviews. All patients were assessed by a qualified child and adolescent psychiatrist within the multidisciplinary team, and diagnoses were established against the DSM-5-TR and ICD-10 criteria in the course of routine specialist care; the limitation therefore lies in the absence of a standardized research instrument such as the K-SADS rather than in the absence of diagnostic criteria. Our operational definition additionally admitted clinically significant symptom clusters documented by the treating team alongside formal diagnoses. The 82.4% figure should accordingly be understood as the prevalence of clinically documented psychiatric burden in this cohort rather than as a research-standard diagnostic prevalence, and it may differ from an instrument-ascertained rate in either direction. Fourth, illness duration prior to admission was not systematically captured, precluding analysis of chronicity as a predictor of hospital course. Fifth, the analyses involved a large number of comparisons without correction for multiplicity. We report unadjusted *p*-values throughout because the comparisons do not form a single pre-specified family and a blanket family-wise correction would misrepresent the design, but this means that findings beyond the pre-specified admission-to-discharge changes should be regarded as hypothesis-generating rather than confirmatory. Relatedly, the partial correlations adjust for admission BMI z-score alone and therefore reduce rather than remove confounding by indication in the caloric analyses; the absence of an independent association between caloric escalation and heart-rate improvement is consistent with, but does not establish, an absence of effect.

## 5. Conclusions

In this consecutive cohort of adolescents admitted for medical stabilization of anorexia nervosa, structured nutritional rehabilitation was associated with significant improvements in weight, BMI z-score, hemodynamic stability, and resolution of bradycardia. Admission BMI z-score was the strongest predictor of length of stay and an important correlate of heart rate recovery, suggesting that baseline nutritional severity remains central to the inpatient course.

Heart rate improved alongside caloric rehabilitation; however, adjusted analyses indicated that baseline nutritional and cardiovascular severity better explained the magnitude of improvement than caloric escalation alone. This finding should be interpreted within the context of a protocolized refeeding program and does not diminish the clinical importance of adequate nutritional rehabilitation for safe and efficient recovery.

The absence of hypophosphatemia requiring intervention or documented refeeding syndrome suggests that the protocol was metabolically safe when delivered with systematic electrolyte monitoring in this specialized tertiary setting. These findings should not be extrapolated to settings with less rigorous monitoring. The high prevalence of psychiatric comorbidity further emphasizes that inpatient care for adolescent anorexia nervosa should begin as an integrated medical, nutritional, and psychiatric intervention, with structured transition to family-supported outpatient recovery.

## Figures and Tables

**Table 1:** Baseline clinical and laboratory characteristics overall and by AN subtype (N = 68). Values are mean ± SD unless stated.

Characteristic	Total (N = 68)	AN-R (*n* = 58)	AN-BP (*n* = 10)
**Demographics**			
Female sex, *n* (%)	62 (91.2%)	53 (91.4%)	9 (90.0%)
Age at admission, years	14.2 ± 1.8	14.3 ± 1.7	14.0 ± 2.4
Prior hospitalization for AN, *n* (%)	3 (4.4%)	3 (5.2%)	0 (0.0%)
Admitted for bradycardia, *n* (%)	53 (77.9%)	45 (77.6%)	8 (80.0%)
**Nutritional status**			
Weight, kg	40.9 ± 8.2	40.8 ± 8.3	41.1 ± 7.9
BMI, kg/m^2^	16.06 ± 2.75	16.02 ± 2.85	16.32 ± 2.16
BMI z-score	−2.16 ± 1.85	−2.24 ± 1.88	−1.71 ± 1.70
BMI%ile †	12.7 ± 20.7	12.5 ± 21.8	14.2 ± 12.6
Admission calories, kcal/day	1231 ± 383	1221 ± 387	1290 ± 373
**Cardiovascular parameters**			
Supine HR, bpm	52.9 ± 10.8	52.2 ± 9.3	56.9 ± 17.3
Standing HR, bpm	63.0 ± 19.3	62.8 ± 18.8	64.1 ± 23.1
Systolic BP, mmHg	91.6 ± 7.4	91.0 ± 7.6	95.2 ± 5.0
Diastolic BP, mmHg *	56.9 ± 6.6	56.2 ± 6.5	61.2 ± 6.1
**Serum electrolytes at admission**			
Sodium, mmol/L	138.0 ± 2.0	138.0 ± 2.1	137.6 ± 1.3
Potassium, mmol/L	4.08 ± 0.39	4.06 ± 0.37	4.14 ± 0.51
Phosphate, mmol/L	1.30 ± 0.16	1.29 ± 0.16	1.33 ± 0.17
Calcium, mmol/L	2.38 ± 0.19	2.37 ± 0.20	2.43 ± 0.11
Magnesium, mmol/L	0.83 ± 0.09	0.83 ± 0.08	0.80 ± 0.13
Bicarbonate, mmol/L	25.5 ± 2.8	25.5 ± 2.6	25.9 ± 4.1
**Hospital course**			
LOS, days (mean ± SD)	36.9 ± 41.6	38.2 ± 41.9	29.0 ± 40.5
LOS, days (median [IQR])	24 [11–41]	25 [12–42]	13 [7–27]

AN-R, restricting type; AN-BP, binge-purge type; BMI, body mass index; † BMI%ile represents the centile position on the WHO BMI-for-age growth reference (range 0–100th centile), derived from the age- and sex-standardized BMI z-score; HR, heart rate; BP, blood pressure; LOS, length of stay; IQR, interquartile range. * Diastolic BP: *p* = 0.023 between subtypes; all other between-subtype comparisons *p* > 0.10. The AN-BP subgroup comprises 10 patients; subtype comparisons should be interpreted with caution.

**Table 2:** Clinical and nutritional parameters at admission and discharge, with change scores (N = 68).

Variable	Admission	Discharge	Mean Change (95% CI)	Median Change [IQR]	*p* †
Weight, kg	40.9 ± 8.2	45.8 ± 7.2	+4.92 (3.95–5.89)	+3.83 [2.0, 7.3]	<0.001
BMI, kg/m^2^	16.06 ± 2.75	18.12 ± 2.48	+2.06 (1.58–2.54)	+1.66 [0.9, 2.6]	<0.001
*BMI z-score*	*−2.16 ± 1.85*	*−0.85 ± 1.14*	*+1.30 (0.99–1.62)*	*+0.91 [0.5, 1.9]*	*<0.001*
BMI%ile	12.7 ± 20.7	26.7 ± 22.5	+13.94 (10.25–17.64)	+10.3 [2.7, 19.7]	<0.001
Supine HR, bpm	52.9 ± 10.8	81.3 ± 13.4	+28.4 (25.0–31.8)	+31 [19, 38]	<0.001
Systolic BP, mmHg	91.6 ± 7.4	100.0 ± 6.6	+8.43 (6.49–10.36)	+7.5 [4.0, 13.3]	<0.001
Diastolic BP, mmHg	56.9 ± 6.6	62.7 ± 6.2	+5.71 (3.80–7.61)	+4.0 [1.0, 11.0]	<0.001
Calories, kcal/day	1231 ± 383	2673 ± 709	+1442 (1242–1642)	+1300 [950, 1850]	<0.001

BMI, body mass index; BMI%ile BMI-for-age percentile (0–100th centile); HR, heart rate; BP, blood pressure; IQR, interquartile range. † All Wilcoxon signed-rank tests also *p* < 0.001.

**Table 3:** Resolution of medical instability criteria from admission to discharge (McNemar’s test; N = 68).

Criterion	Admission *n* (%)	Discharge *n* (%)	Improved *n*	Worsened *n*	McNemar *p*
HR < 50 bpm	27 (39.7%)	1 (1.5%)	26	0	<0.001
SBP < 90 mmHg	22 (32.4%)	2 (2.9%)	21	1	<0.001
BMI < 15 kg/m^2^	24 (35.3%)	5 (7.4%)	19	0	<0.001
BMI z-score < −2	28 (41.2%)	10 (14.7%)	18	0	<0.001

HR, heart rate; BP, blood pressure; BMI, body mass index.

**Table 4:** Spearman rank correlations with length of stay (N = 68), ordered by absolute ρ.

Predictor	Spearman ρ	*p* Value
Weight change, kg	0.682	<0.001
BMI z-score change	0.656	<0.001
BMI change, kg/m^2^	0.644	<0.001
Admission BMI z-score	−0.472	<0.001
Admission BMI%ile	−0.467	<0.001
Admission BMI, kg/m^2^	−0.458	<0.001
Calorie change, kcal/day	0.427	<0.001
BMI%ile change	0.379	0.001
Admission weight, kg	−0.346	0.004
Admission calories, kcal/day	−0.207	0.090
HR change, bpm	0.152	0.215
Admission systolic BP, mmHg	−0.149	0.225
Admission diastolic BP, mmHg	−0.139	0.257
Age at admission, years	0.096	0.438
Admission supine HR, bpm	0.014	0.910

BMI, body mass index; BMI%ile; BMI-for-age percentile (0–100th centile); HR, heart rate; BP, blood pressure.

**Table 5:** Bivariate and partial correlations between caloric intake and heart-rate parameters (N = 68).

Analysis	N	Pearson r	*p*	Spearman ρ	*p*	Partial r (Adj. BMI z-Score)
*Primary cohort (N = 68)*						
Admission cal vs. admission HR	68	0.324	0.007	0.182	0.138	0.297 (*p* = 0.014)
Discharge cal vs. discharge HR	68	0.150	0.222	0.181	0.140	0.107 (*p* = 0.388)
Calorie increase vs. HR improvement	68	0.153	0.214	0.173	0.159	0.052 (*p* = 0.674)
Discharge cal vs. HR improvement	68	0.114	0.356	0.139	0.258	0.025 (*p* = 0.843)
*Sensitivity: excluding admission calories < 100 kcal/day (n = 67)*						
Admission cal vs. admission HR	67	0.462	<0.001	0.256	0.036	0.437 (*p* < 0.001)
Calorie increase vs. HR improvement	67	0.208	0.091	0.205	0.096	0.108 (*p* = 0.387)

Partial correlations control for admission BMI z-score. HR, heart rate; cal, caloric intake; SE, standard error; CI, confidence interval.

**Table 6:** Multivariable linear regression predicting heart-rate improvement (discharge minus admission supine HR) and discharge HR (N = 68).

Predictor	β	SE	95% CI	*p* Value	Sig.
**Model A: HR improvement (R^2^ = 0.319, adjusted R^2^ = 0.252)**					
Calorie change per 1000 kcal	−0.48	2.00	−4.48 to +3.52	0.812	ns
Admission BMI z-score	−2.70	0.98	−4.65 to −0.74	0.008	**
Admission supine HR	−0.476	0.145	−0.767 to −0.186	0.002	**
Age at admission, years	−2.13	0.92	−3.97 to −0.29	0.024	*
Male sex	−7.91	5.98	−19.87 to +4.04	0.190	ns
AN binge-purge type	+2.69	4.26	−5.82 to +11.21	0.529	ns
**Model B: Discharge HR (R^2^ = 0.260, adjusted R^2^ = 0.174)**					
Discharge calories per 1000 kcal	−0.08	2.34	−4.76 to +4.61	0.974	ns
Admission BMI z-score	−2.42	0.99	−4.41 to −0.43	0.018	*
Admission supine HR	+0.510	0.148	+0.214 to +0.806	0.001	**
Age at admission, years	−2.11	0.93	−3.97 to −0.25	0.027	*
Male sex	−7.28	6.04	−19.37 to +4.80	0.233	ns
AN binge-purge type	+3.04	4.28	−5.53 to +11.60	0.481	ns

HR, heart rate; SE, standard error; CI, confidence interval. Sig.: * *p* < 0.05; ** *p* < 0.01; ns, not significant. Both models additionally adjusted for LOS (LOS was not an independent predictor in either model; *p* > 0.36).

**Table 7:** Medical and mental health comorbidity prevalence overall and by AN subtype (N = 68).

Comorbidity	Overall *n* (%)	AN-R *n* (%)	AN-BP *n* (%)	*p* †
Any psychiatric comorbidity	56 (82.4%)	47 (81.0%)	9 (90.0%)	0.678
Depression	40 (58.8%)	34 (58.6%)	6 (60.0%)	1.000
Anxiety disorder	29 (42.6%)	24 (41.4%)	5 (50.0%)	0.733
Amenorrhea (primary/secondary)	19 (27.9%)	17 (29.3%)	2 (20.0%)	0.714
Suicidal ideation or attempt	13 (19.1%)	12 (20.7%)	1 (10.0%)	0.673
Self-harm behavior	12 (17.6%)	10 (17.2%)	2 (20.0%)	1.000
Autism spectrum disorder	5 (7.4%)	5 (8.6%)	0 (0.0%)	1.000
Obsessive-compulsive disorder	4 (5.9%)	4 (6.9%)	0 (0.0%)	1.000
ADHD	2 (2.9%)	1 (1.7%)	1 (10.0%)	0.274

ADHD, attention-deficit/hyperactivity disorder; AN-R, restricting type; AN-BP, binge-purge type. † Fisher’s exact test. All between-subtype comparisons *p* > 0.05. AN-BP subgroup *n* = 10; subtype comparisons should be interpreted with caution.

## Data Availability

De-identified data supporting the reported results are available from the corresponding author upon reasonable request, subject to institutional data-sharing agreements and patient privacy regulations.

## Supplementary Materials

The following supporting information can be downloaded at: https://www.mdpi.com/article/10.3390/nu18172789/s1, Supplementary Material S1: STROBE Checklist for Cohort Studies. Supplementary Material S2: Cohort Eligibility and Patient Flow. Supplementary Material S3: Operational Definitions. Supplementary Material S4: Nutritional Rehabilitation and Monitoring Protocol. Supplementary Material S5: Supplementary Figures. Table S1 summarizes cohort eligibility and inclusion for the retrospective consecutive inpatient cohort. Table S2: Operational definitions. Table S3: Inpatient nutritional rehabilitation protocol. Table S4: Electrolyte monitoring and refeeding safety definitions. Figure S1: Admission BMI z-score and length of stay (LOS) in days (N = 68). Figure S2: Admission BMI-for-age percentile and length of stay (LOS) in days (N = 68). Figure S3: Weight gain during admission and length of stay (LOS) in days (N = 68). Figure S4: Calorie increase from admission to discharge and heart-rate improvement: sensitivity analysis. Figure S5: Discharge calories and discharge heart rate in 68 adolescents with anorexia nervosa. Figure S6: Admission calories and admission lowest heart rate in 68 adolescents with anorexia nervosa.
