# Supplementary material for: Nutritional Rehabilitation and Medical Stabilization of Adolescents with Anorexia Nervosa: Length-of-Stay Predictors, Cardiovascular Recovery, Refeeding Safety, and Psychiatric Comorbidity Burden
Source: Nutrients. 2026 Aug 26;18(17):2789. doi: 10.3390/nu18172789 (PMC13567540; doi:10.3390/nu18172789)
Supplement: Supplementary file 1 [file nutrients-18-02789-s001.zip › nutrients-4440354-supplementary.pdf]

Supplementary Materials

Nutritional Rehabilitation and Medical Stabilization of Adolescents with Anorexia Nervosa: Length-of-Stay Predictors, Cardiovascular Recovery, Refeeding Safety, and Psychiatric Comorbidity Burden

Article type: Original Article. Special Issue: Advances in Eating Disorders: Nutritional Perspectives.

Supplementary Material S1. STROBE Checklist for Cohort Studies

The completed STROBE checklist below identifies where each reporting item is addressed in the manuscript and supplementary materials.

Section/Topic	Item	STROBE recommendation	Location in manuscript
Title and abstract	1a	Indicate the study design with a commonly used term in the title or the abstract.	Title and Abstract
Title and abstract	1b	Provide in the abstract an informative and balanced summary of what was done and what was found.	Abstract
Introduction: Background/rationale	2	Explain the scientific background and rationale for the investigation being reported.	Introduction
Introduction: Objectives	3	State specific objectives, including any prespecified hypotheses.	End of Introduction
Methods: Study design	4	Present key elements of study design early in the paper.	Methods, Study Design, Setting, and Participants
Methods: Setting	5	Describe the setting, locations, and relevant dates, including periods of recruitment, exposure, follow-up, and data collection.	Methods, Study Design, Setting, and Participants
Methods: Participants	6a	Give eligibility criteria, and the sources and methods of selection of participants.	Methods, Study Design, Setting, and Participants; Supplementary Table S1
Methods: Participants	6b	For matched studies, give matching criteria and numbers of exposed and unexposed.	Not applicable; this was not a matched cohort study
Methods: Variables	7	Clearly define all outcomes, exposures, predictors, potential confounders, and effect modifiers.	Methods, Clinical Variables and Outcome Measures; Supplementary Table S2
Methods: Data sources/measurement	8	For each variable of interest, give sources of data and details of methods of assessment/measurement.	Methods, Clinical Variables and Outcome Measures; Supplementary Tables S2-S4
Methods: Bias	9	Describe any efforts to address potential sources of bias.	Methods and Discussion; consecutive cohort design, uniform protocol, adjusted models, and sensitivity analyses
Methods: Study size	10	Explain how the study size was arrived at.	Methods, Study Design, Setting, and Participants; all eligible consecutive admissions during the study period were included
Methods: Quantitative variables	11	Explain how quantitative variables were handled in the analyses, including any groupings.	Methods, Clinical Variables and Outcome Measures; Statistical Analysis
Methods: Statistical methods	12a	Describe all statistical methods, including those used to control for confounding.	Methods, Statistical Analysis
Methods: Statistical methods	12b	Describe any methods used to examine subgroups and interactions.	Methods and Results; anorexia nervosa subtype comparisons were reported descriptively

Section/Topic	Item	STROBE recommendation	Location in manuscript
Methods: Statistical methods	12c	Explain how missing data were addressed.	Methods; complete admission and discharge clinical data were required for inclusion; available psychiatric data were abstracted for all included patients
Methods: Statistical methods	12d	If applicable, explain how loss to follow-up was addressed.	Not applicable to the primary inpatient admission-to-discharge outcomes
Methods: Statistical methods	12e	Describe any sensitivity analyses.	Methods, Statistical Analysis; Results
Results: Participants	13a	Report numbers of individuals at each stage of study.	Results, Baseline Characteristics; Supplementary Table S1
Results: Participants	13b	Give reasons for non-participation at each stage.	Methods and Supplementary Table S1; eligibility and exclusion criteria are stated
Results: Participants	13c	Consider use of a flow diagram.	Supplementary Table S1 provides a cohort eligibility summary
Results: Descriptive data	14a	Give characteristics of study participants and information on exposures and potential confounders.	Results, Table 1
Results: Descriptive data	14b	Indicate number of participants with missing data for each variable of interest.	Methods and Results; final analytic cohort required complete admission and discharge clinical data
Results: Descriptive data	14c	Summarize follow-up time.	Not applicable; no post-discharge follow-up outcome was analyzed; inpatient LOS is reported
Results: Outcome data	15	Report numbers of outcome events or summary measures over time.	Results, Tables 2-3
Results: Main results	16a	Give unadjusted estimates and, if applicable, confounder-adjusted estimates and their precision.	Results, Tables 4-6
Results: Main results	16b	Report category boundaries when continuous variables were categorized.	Methods, Clinical Variables and Outcome Measures; Results, medical instability criteria
Results: Main results	16c	If relevant, consider translating estimates of relative risk into absolute risk for a meaningful time period.	Not applicable; the study reports continuous and in-hospital outcomes rather than risk over time
Results: Other analyses	17	Report other analyses performed, including subgroup and sensitivity analyses.	Results
Discussion: Key results	18	Summarize key results with reference to study objectives.	Discussion
Discussion: Limitations	19	Discuss limitations of the study.	Discussion, limitations paragraph
Discussion: Interpretation	20	Give a cautious overall interpretation of results considering objectives, limitations, multiplicity, and evidence from similar studies.	Discussion and Conclusions
Discussion: Generalizability	21	Discuss the generalizability of the study results.	Discussion
Other information: Funding	22	Give the source of funding and the role of funders for the present study.	Funding statement

Supplementary Material S2. Cohort Eligibility and Patient Flow

Table S1 summarizes cohort eligibility and inclusion for the retrospective consecutive inpatient cohort.

Stage	Definition / source	Number / status
Source population	Adolescents aged 8-17 years admitted for inpatient medical stabilization of anorexia nervosa at the national tertiary pediatric eating disorders program between January 2017 and May 2025.	Consecutive eligible admissions identified from clinical records
Eligibility criteria	Confirmed DSM-5-TR diagnosis of anorexia nervosa; admission for medical stabilization; complete admission and discharge clinical data; age <=18 years at admission.	Applied to all included records
Exclusion criteria	Alternative primary diagnosis, incomplete admission records, or hospital stay <24 hours.	Excluded records were not retained in the final analytic dataset
Final analytic cohort	Consecutive adolescents meeting all inclusion criteria during the study period.	68
Prior hospitalization for AN-related instability	Documented prior admission within five years of the index admission.	3/68 (4.4%)

Supplementary Material S3. Operational Definitions

Table S2: Operational definitions

Domain	Variable / criterion	Operational definition
Diagnosis	Anorexia nervosa	DSM-5-TR diagnosis established by the treating physician and/or multidisciplinary eating-disorder team.
Diagnosis	Anorexia nervosa subtype	Classified as restricting type or binge-purge type according to clinical documentation.
Nutritional status	BMI	Weight in kilograms divided by height in metres squared.
Nutritional status	BMI z-score	Age- and sex-standardized BMI z-score using WHO reference standards, as stated in the manuscript.
Nutritional status	BMI-for-age percentile	Recorded in the source dataset as %mBMI and analyzed in the manuscript as a 0-100th BMI-for-age centile variable. In this manuscript, this variable is not used to mean percent median BMI.
Hospital course	Length of stay	Number of days between admission and discharge from the index inpatient admission.
Cardiovascular	Supine heart rate	Resting supine heart rate in beats per minute.
Medical instability	Bradycardia	Supine HR <50 bpm.

Domain	Variable / criterion	Operational definition
Medical instability	Hypotension	Systolic blood pressure <90 mmHg.
Medical instability	Low absolute BMI	BMI <15 kg/m2.
Medical instability	Low BMI z-score	BMI z-score <-2.
Electrolyte safety	Hypophosphatemia	Serum phosphate <0.81 mmol/L.
Electrolyte safety	Severe hypophosphatemia	Serum phosphate <0.60 mmol/L.
Psychiatric comorbidity	Any psychiatric comorbidity	At least one documented concurrent psychiatric diagnosis or clinically significant symptom cluster in treating psychiatrist documentation.

Supplementary Material S4. Nutritional Rehabilitation and Monitoring Protocol

Table S3. Inpatient nutritional rehabilitation protocol.

Component	Protocol detail
Initial caloric prescription	Patients consuming <=500 kcal/day before admission were commenced at 1,000 kcal/day; those consuming >500 kcal/day were commenced at 1,200 kcal/day.
Caloric escalation	Calories were advanced by 200 kcal/day every 48 hours, with clinical adjustments according to weight-gain trajectory, electrolyte values, and individual tolerance.
Target discharge intake	2,500-3,000 kcal/day divided into three meals and two to three snacks.
Meal supervision	All meals were supervised by nursing staff.
Oral supplementation	If a meal was not completed within 45 minutes, the shortfall was supplemented using Pediasure 1.5 kcal/mL.
Nasogastric feeding	Used if oral or supplement intake remained insufficient; required in approximately 1-2 patients for 1-2 days each before oral intake was resumed.
Micronutrients	Children's multivitamin prescribed throughout admission.
Activity	Bed rest initially, with graduated mobilization as cardiovascular parameters normalized.

Table S4. Electrolyte monitoring and refeeding safety definitions.

Parameter	Protocol / definition / observed result
Monitoring window	Serum electrolytes, including phosphate and magnesium, were measured daily from day 2 through day 7 of admission.
Reduced monitoring frequency	If values remained within normal limits, testing was reduced to every 48-72 hours or as clinically indicated.
Replacement approach	If hypophosphatemia or hypomagnesemia occurred, oral or intravenous replacement was provided and daily monitoring continued until normalization.
Routine prophylactic phosphate	Not routinely administered to patients with normal admission electrolytes.
Admission phosphate	Mean 1.30 +/- 0.16 mmol/L; no admission hypophosphatemia reported.
Refeeding hypophosphatemia	No hypophosphatemia requiring intervention was documented during the monitored refeeding period.
Refeeding syndrome	No case of refeeding syndrome was documented during admission.
Most frequent admission abnormality	Hypokalemia was present in 5/68 patients (7.4%) at admission.

Supplementary Material S5. Supplementary Figures

Supplementary figures provide visualizations supporting the LOS, nutritional rehabilitation, and heart-rate recovery analyses reported in the main manuscript.

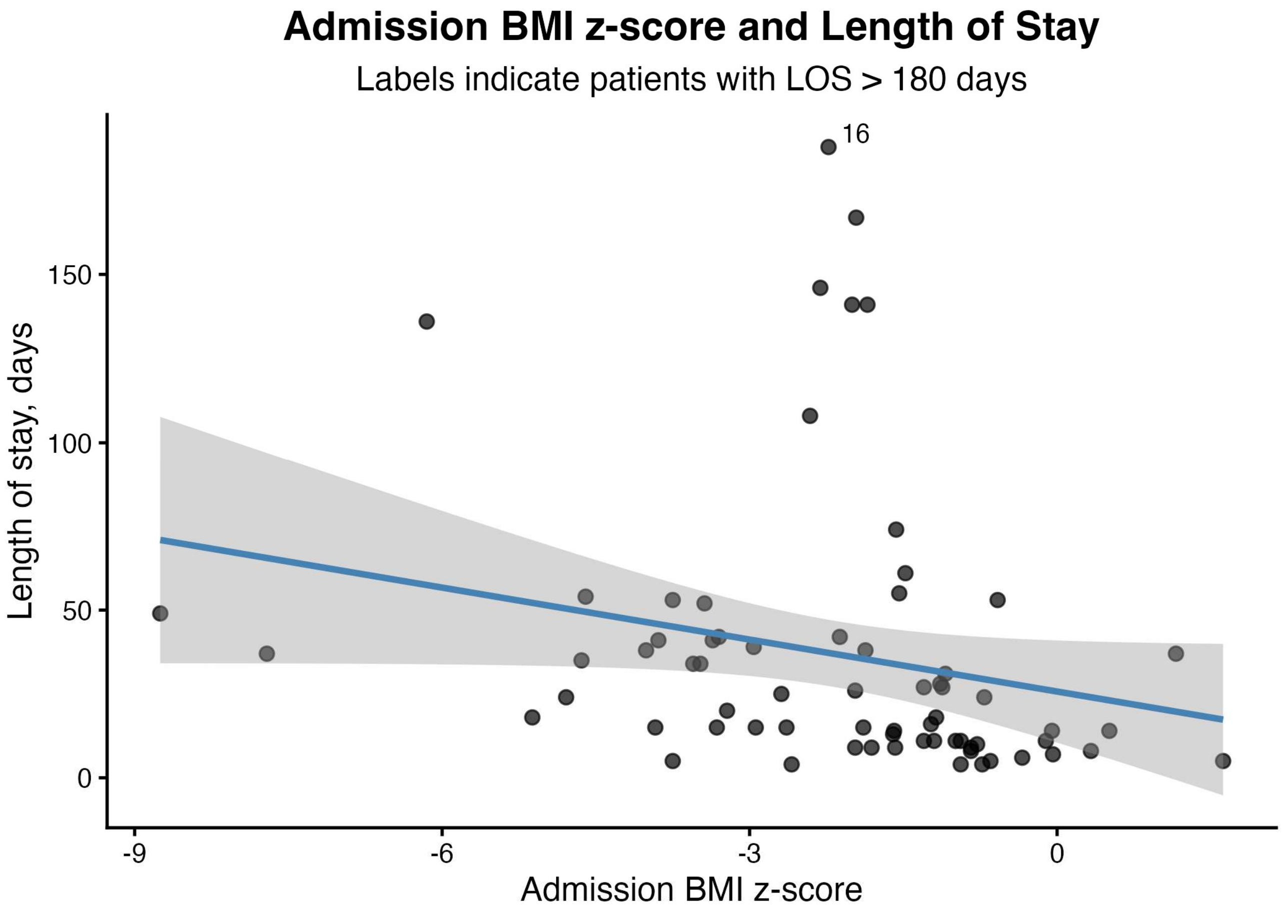

Figure S1. Admission BMI z-score and length of stay (LOS) in days (N = 68). Each point represents one patient; the label indicates the patient with LOS >180 days (LOS = 188 days). The regression line and 95% confidence band illustrate the inverse association: more severely malnourished patients (lower BMI z-score) had significantly longer admissions (Spearman rho = -0.472, p < 0.001). In log-LOS multivariable regression adjusted for age, sex, and AN subtype, each 1-unit increment in admission BMI z-score was associated with an 18.9% shorter LOS (beta = -0.209, 95% CI -0.340 to -0.076; p = 0.003). The scatter plot displays raw LOS for visualization, whereas all inferential analyses used log-transformed LOS.

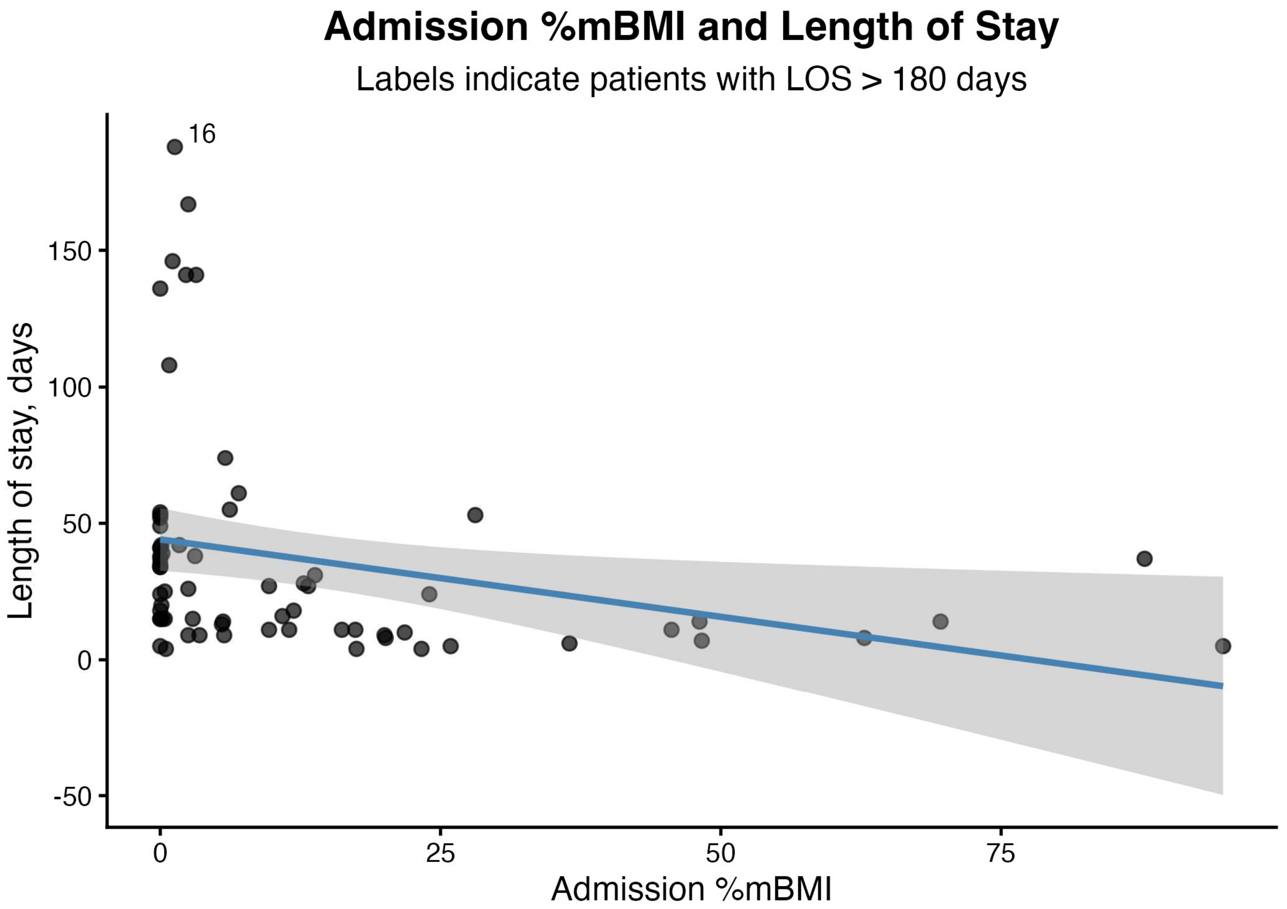

Figure S2. Admission BMI-for-age percentile and length of stay (LOS) in days (N = 68). The x-axis label in the source figure uses %mBMI, which denotes the source-data BMI-for-age percentile variable (0-100th centile) and should not be interpreted as percent median BMI. Labels indicate patients with LOS >180 days. The inverse association between BMI-for-age percentile and LOS was significant (Spearman rho = -0.467, $p < 0.001$); in multivariable regression, each 1 percentile-point increment was associated with a 1.7% shorter LOS (beta = -0.018, 95% CI -0.028 to -0.007; $p = 0.002$; $R2 = 0.170$).

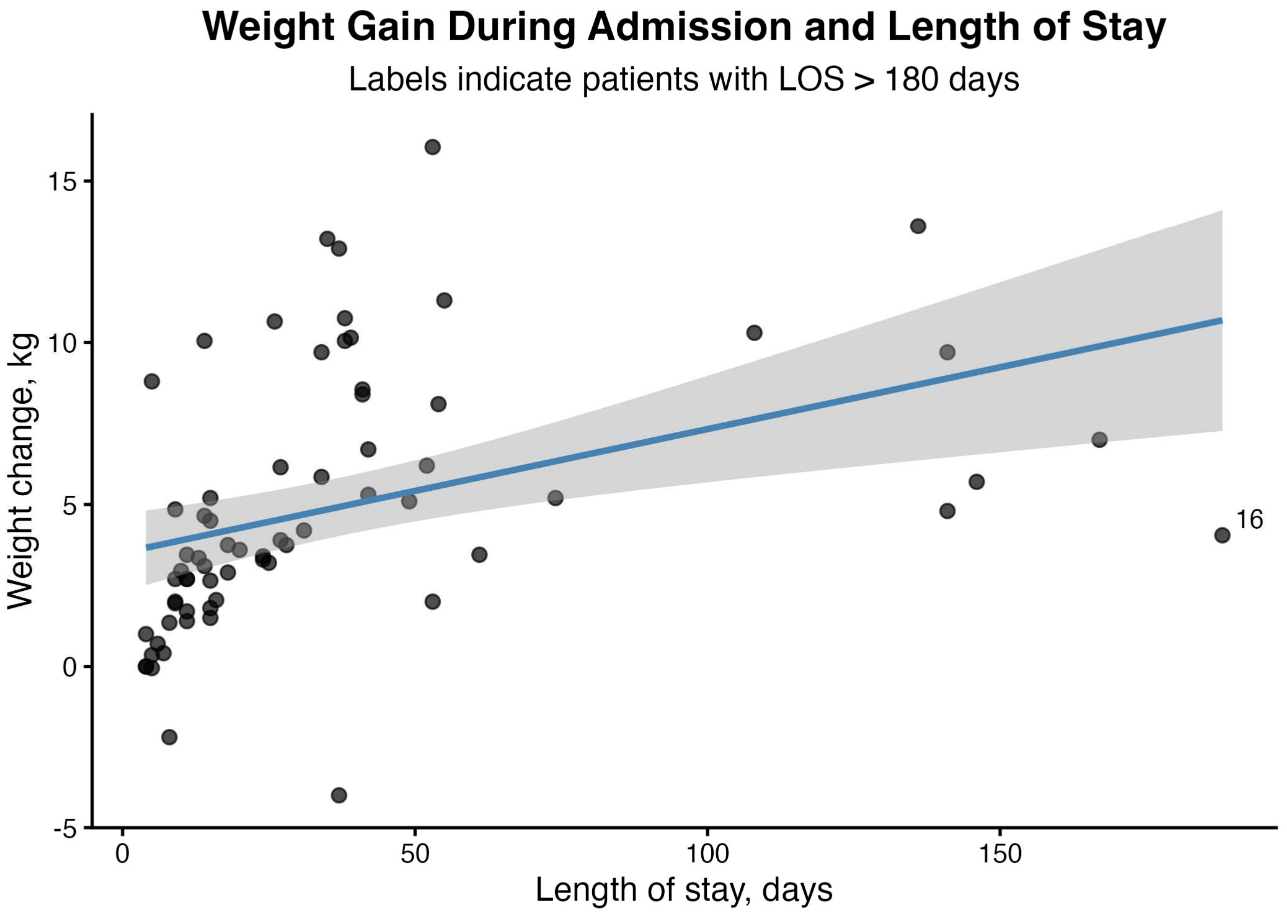

Figure S3. Weight gain during admission and length of stay (LOS) in days (N = 68). The positive association (Spearman rho = 0.682, $p < 0.001$) reflects that patients who remained hospitalized longer achieved greater total weight restoration, as expected in a structured nutritional rehabilitation pathway. This correlation describes the cumulative outcome of the inpatient rehabilitation process and should not be interpreted as evidence that longer LOS independently caused faster weight-gain rate.

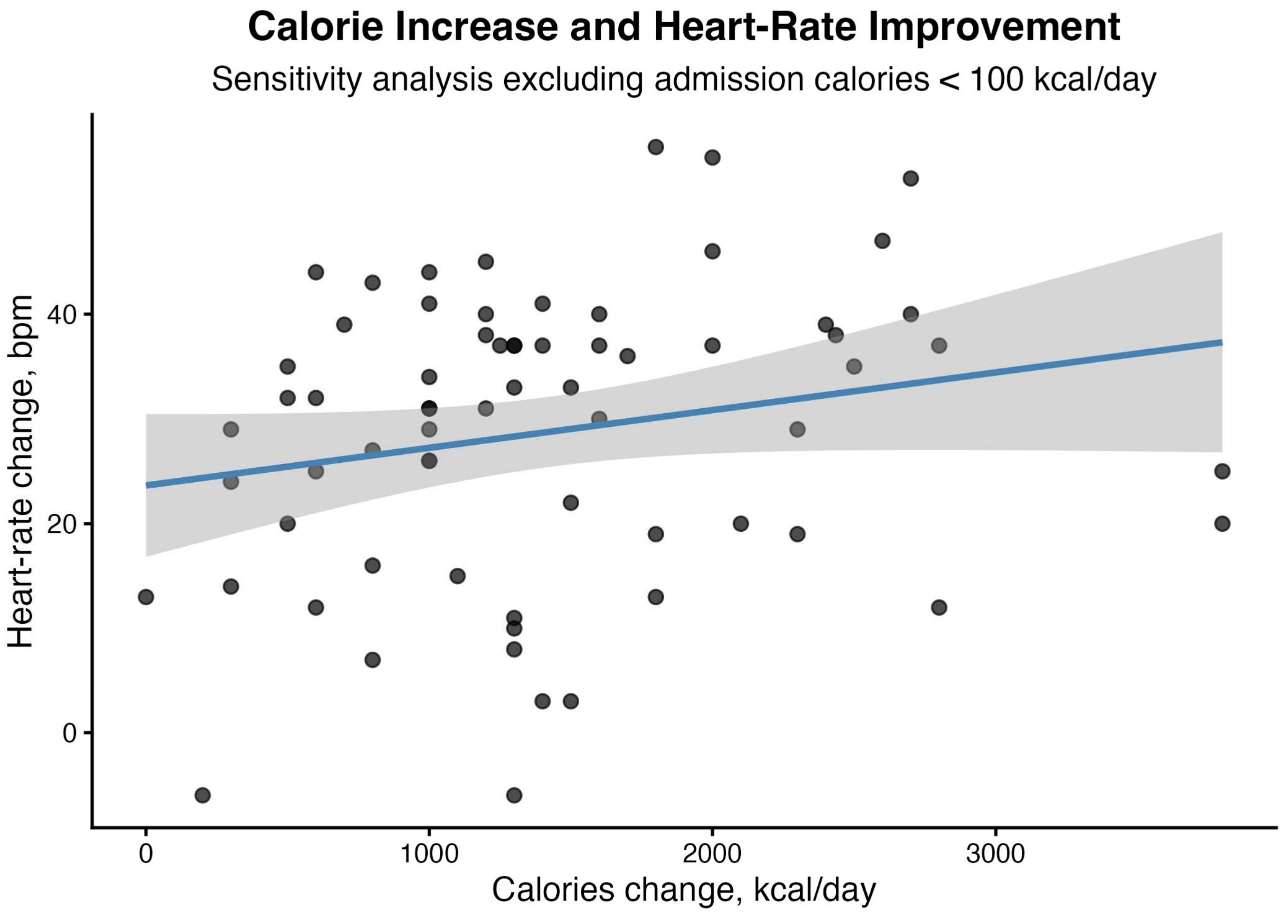

Figure S4. Calorie increase from admission to discharge and heart-rate improvement: sensitivity analysis. This analysis excludes the patient with admission calories <100 kcal/day (n = 67). The association between caloric escalation and HR improvement remained non-significant after controlling for admission BMI z-score (partial r = 0.108, p = 0.387), supporting that the primary finding was not driven by this outlier.

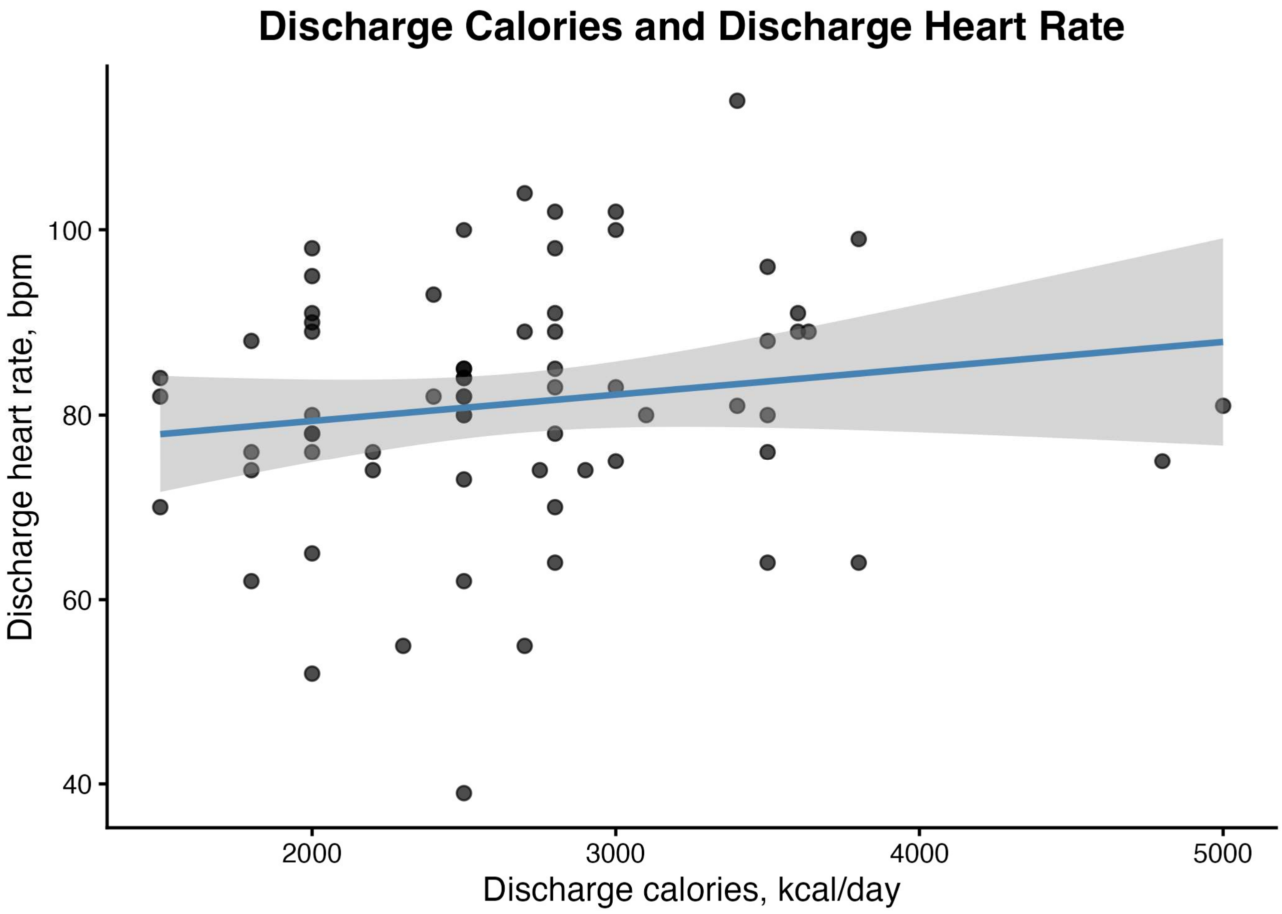

Figure S5. Discharge calories and discharge heart rate in 68 adolescents with anorexia nervosa. No significant association was observed between caloric prescription at discharge and discharge HR (Pearson r = 0.150, p = 0.222; partial r controlling for admission BMI z-score = 0.107, p = 0.388).

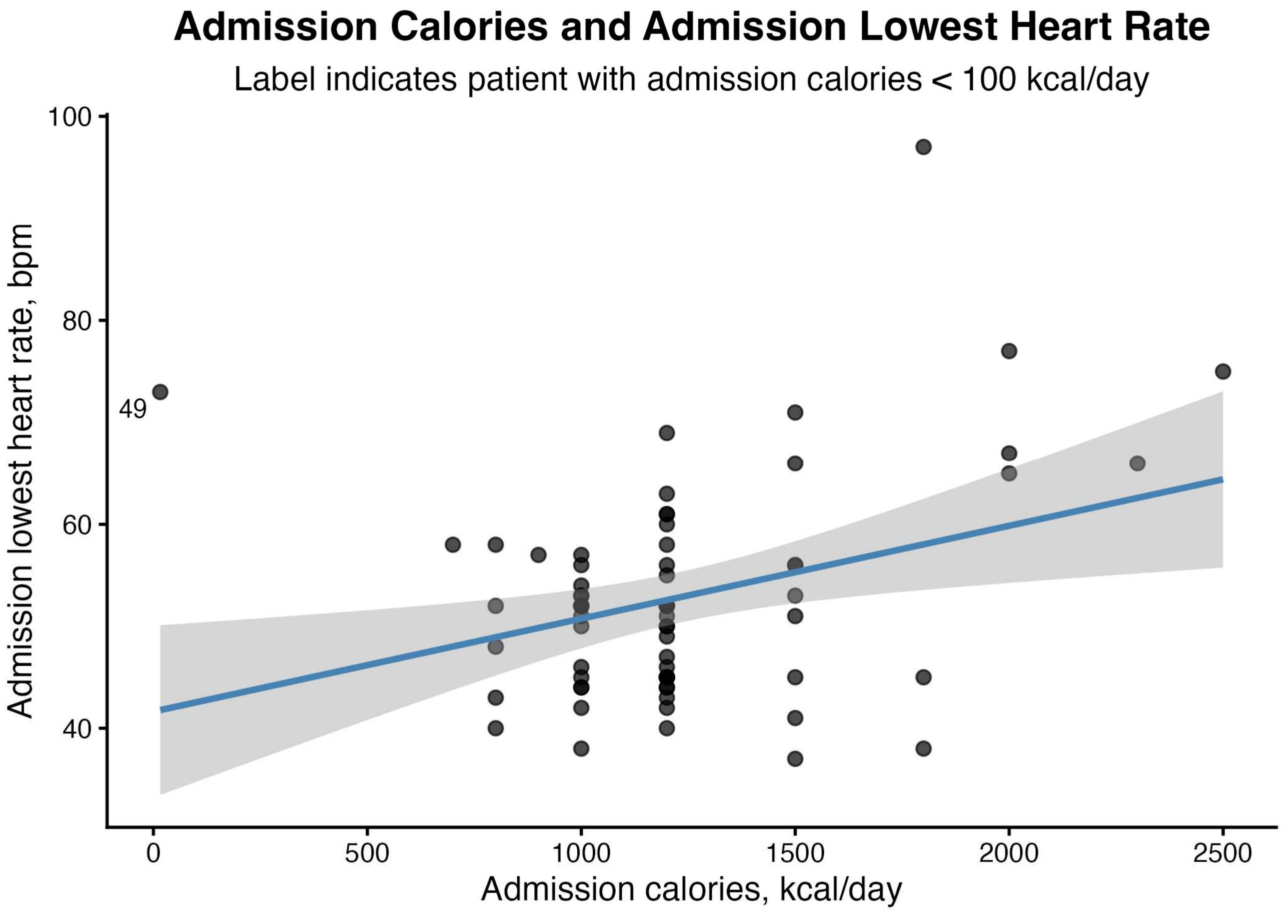

Figure S6. Admission calories and admission lowest heart rate in 68 adolescents with anorexia nervosa. The label indicates the patient with admission calories <100 kcal/day. Admission caloric intake was positively associated with admission HR in the primary cohort (Pearson r = 0.324, p = 0.007; partial r controlling for admission BMI z-score = 0.297, p = 0.014). After exclusion of the low-admission-calorie outlier, this association strengthened (partial r = 0.437, p < 0.001). This cross-sectional association reflects baseline clinical severity rather than a causal effect of calories on HR.
